# Sugar sweetened beverages, natural fruit juices, and cancer: what we know and what still needs to be assessed

**DOI:** 10.3389/fnut.2023.1301335

**Published:** 2023-12-21

**Authors:** Niloofar Eshaghian, Mohammad Javad Zare, Mohammad Keshavarz Mohammadian, Zohre Gozidehkar, Afsaneh Ahansaz, Gholamreza Askari, Masoomeh Asadi, Alireza Milajerdi, Omid Sadeghi

**Affiliations:** ^1^Student Research Committee, Isfahan University of Medical Sciences, Isfahan, Iran; ^2^Nutrition Research Center, School of Nutrition and Food Sciences, Shiraz University of Medical Sciences, Shiraz, Iran; ^3^Department of Nutrition, Science and Research Branch, Islamic Azad University, Tehran, Iran; ^4^Department of Public Health, Birjand University of Medical Sciences, Birjand, Iran; ^5^Department of Food Science and Technology, Faculty of Agriculture, University of Tabriz, Tabriz, Iran; ^6^Nutrition and Food Security Research Center, Department of Community Nutrition, School of Nutrition and Food Science, Isfahan University of Medical Sciences, Isfahan, Iran; ^7^Department of Operating Room Nursing, Abadan University of Medical Sciences, Abadan, Iran; ^8^Research Center for Biochemistry and Nutrition in Metabolic Diseases, Institute for Basic Sciences, Kashan University of Medical Sciences, Kashan, Iran

**Keywords:** sugar-sweetened beverages, natural fruit juices, carbonated beverages, cancer, cancer mortality

## Abstract

Cancer is known as one of the leading causes of death in the world. In addition to early mortality, cancer is associated with disability in affected patients. Among environmental risk factors, special attention has been paid to the role of dietary factors. In recent decades, the consumption of sugar-sweetened beverages (SSBs) and natural fruit juices has increased. Several studies have assessed the effects of these beverages on human health and found that a higher intake of SSBs is associated with a greater risk of obesity, diabetes, cardiovascular diseases, hypertension, and non-alcoholic fatty liver disease. However, current evidence for cancer incidence and mortality is not conclusive. In the current review, we concluded that SSBs intake might be positively associated with cancer incidence/mortality through their increasing effects on obesity, inflammatory biomarkers, serum levels of insulin-like growth factor-I (IGF-I), and advanced glycation end-products. Such a positive association was also seen for natural fruit juices. However, types of natural fruit juices were not considered in most previous studies. In addition, some types of cancer including brain, lung, and renal cancers were not assessed in relation to SSBs and natural fruit juices. Therefore, further studies are needed in this regard.

## Introduction

Cancer is known as one of the leading causes of death in the world (1). In 2020, 19.3 million new cases of cancer and approximately 10 million cancer deaths occurred worldwide (2). In addition to early mortality, cancer is associated with disability in affected patients and imposes a high economic burden on the health care system (3). Therefore, finding approaches to prevent cancer and cancer-related mortality is necessary.

It is well known that genetic and environmental factors such as smoking, low physical activity, and unhealthy diet are involved in cancer etiology. Previous studies have shown that several dietary factors have a potential role in cancer incidence and mortality (4). For instance, higher intake of red and processed meats was associated with an increased risk of cancer and cancer-related mortality (5), while higher consumption of fruits and vegetables was associated with a reduction in cancer incidence (6), and mortality (7). In recent decades, the intake of sweetened beverages, including sugar-sweetened beverages (SSBs) and fruit juices, and their associations with cancer received much attention. SSB consumption has increased worldwide, especially among adolescents (8). SSBs are beverages containing caloric sweeteners (sucrose, fructose, etc.), which include carbonated drinks, soft drinks, and fruit drinks.

**Figure 1:**
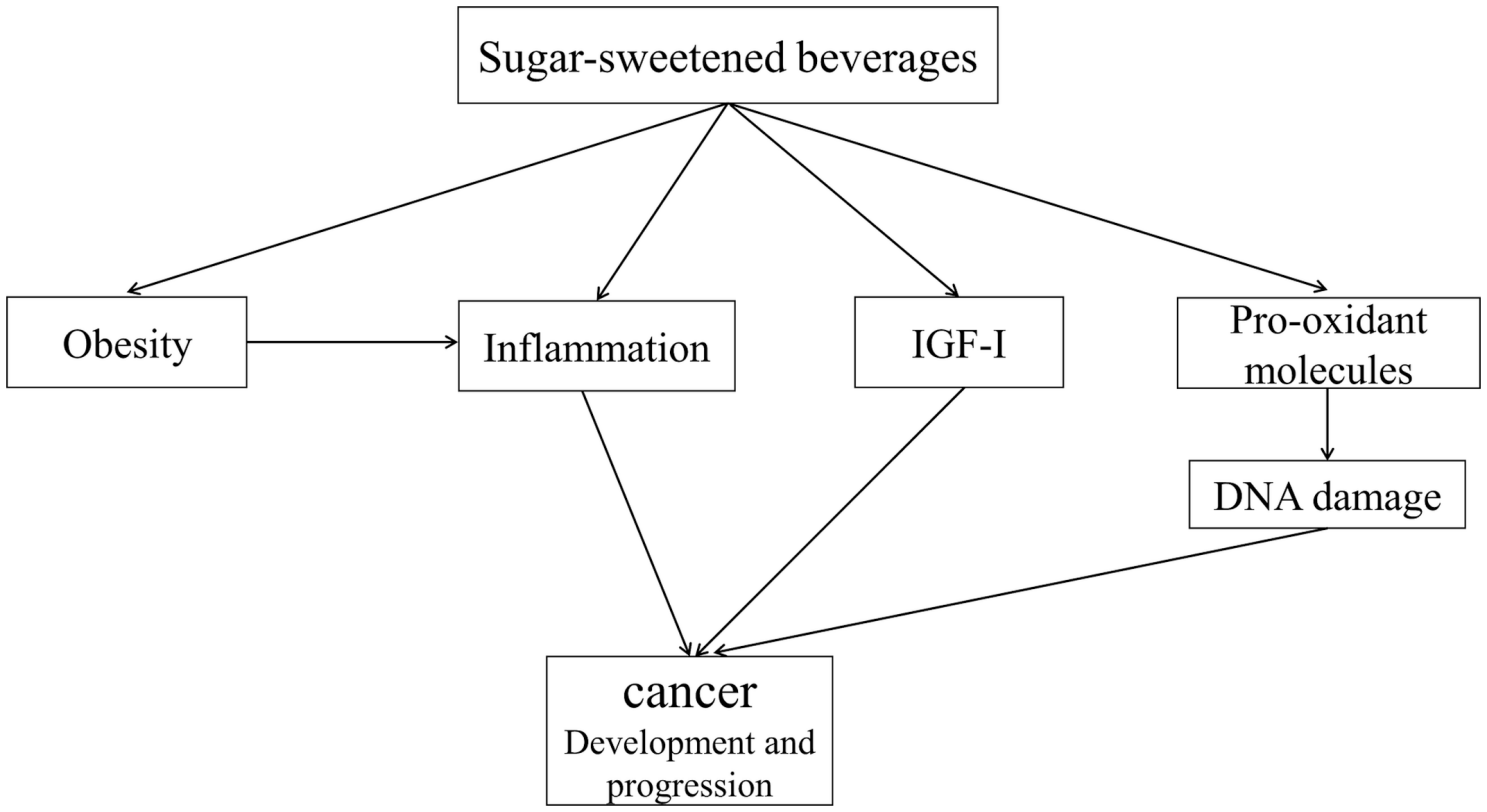
Mechanisms involved in the influence of cancer development and progression. Higher intake of SSBs is associated with obesity, which is associated with increased levels of inflammatory biomarkers, particularly IL-6. IL-6 plays an important role in the proliferation and differentiation of cells. Excessive consumption of sugar from SSBs leads to visceral fat accumulation. Compared to subcutaneous fat, visceral fat induces a high level of pro-inflammatory activity and drives a systemic proinflammatory environment, which is associated with cancer development and progression. Also, high sugar intake increases postprandial blood glucose, which in turn stimulates the production of pro-oxidant molecules and induces DNA damage, thereby increasing cancer risk. Furthermore, SSBs consumption is associated with hyperinsulinemia and higher levels of IGF-I, which might be associated with cancer progression. SSBs: sugar-sweetened beverages, IL-6: interleukin-6, IGF-I: circulating insulin-like growth factor-I.

There is considerable evidence linking excessive consumption of SSBs to a wide range of health problems. Several studies have shown that consumption of SSBs was associated with a higher risk of type 2 diabetes mellitus (9), hypertension, cardiometabolic diseases (10, 11), non-alcoholic fatty liver disease (12), and inflammatory disorders (13–15). The positive association between SSBs and obesity risk has also been reported (16, 17). In terms of cancer, it has been shown that SSBs might affect cancer risk through chronic inflammation and hormonal imbalance (18–20). There might also be an indirect association between SSBs and cancer risk through obesity. Furthermore, SSBs consumption is associated with glycemic response, hyperinsulinemia, and higher levels of circulating insulin-like growth factor-I (IGF-I), which might be associated with cancer progression (21, 22) (Figure 1). Despite the mentioned evidence, findings from epidemiological studies on the association between the consumption of sweetened beverages and the risk of cancer and its mortality are controversial. In this review, we aimed to summarize the epidemiological evidence on the associations of sweetened beverages in different types with cancer incidence/mortality in adults and also discuss the controversial findings in this regard.

## Methods

In this review article, we conducted an electronic search in the online databases of PubMed, ISI Web of Science, and Scopus to identify eligible articles between July 2000 and July 2023. The following search terms were used in the search strategy: (“sugar-sweetened beverage” OR “sweetened carbonated beverage” OR “sweetened beverage” OR “sugary drink” OR “sugary beverages” OR “sugar-sweetened soft drinks”) AND (“cancer” OR “cancer mortality”). We included observational studies and meta-analyses of observational studies that examined the association of consumption of SSBs and natural fruit juices with cancer incidence/mortality in adults. In the current study letters, comments, and animal studies were excluded. Finally, 32 articles were included in our study.

### Definition of SSBs

At present, there is no universal consensus on the definition of SSBs. However, the most accepted definition is to consider any beverage as an SSB if it contains caloric sweeteners such as high-fructose corn syrup (HFCS), sucrose, or fruit juice concentrates among others, which are added to the beverages by individuals, establishments, or manufacturers. According to the definition of the New York City Board of Health, SSBs are considered beverages that have ≥25 calories or 6.25 g of added sugar per 237 mL (23). Unsweetened fruit juices are not considered SSBs, since the sugars in these beverages are not added and are naturally occurring.

### Sweetened drinks and cancer incidence/mortality

Below, we summarized available findings on each type of sweetened drinks (carbonated and non-carbonated drinks and soda) in relation to cancer and then, explained the controversial findings and the possible mechanisms for the associations. We also explained natural fruit juices and their relationship with cancer and cancer mortality.

### Non-carbonated SSBs

All types of beverages with added sugar are classified as SSBs and include carbonated and non-carbonated soft drinks, energy drinks, sports drinks, industrial fruit juices, sweetened coffee and tea, and other drinks with added caloric sweeteners such as HFCS and sucrose (24). The rate of SSBs consumption differs in each region of the world (25). Because of the widespread consumption of SSBs, there are several studies that examined SSBs intake in relation to health outcomes (26). The higher intake of SSBs was associated with type 2 diabetes, non-alcoholic fatty liver disease, depression, coronary heart disease, stroke, cardiovascular diseases, and all-cause mortality (27–29). Moreover, higher intake of SSBs was associated with obesity, insulin resistance, and changes in body composition (30–32). Therefore, these associations make a question of whether there is any association between SSBs intake cancer incidence or mortality.

A meta-analysis of 21 prospective cohort studies showed that a higher consumption of SSBs was associated with a 10% increased risk of total cancer (relative risk (RR): 1.10, 95% confidence interval (CI): 1.03–1.17), and a 6% increased risk of cancer mortality (RR: 1.06, 95% CI: 1.01–1.12). In that meta-analysis, the dose–response association was also assessed, in which each 250 mL increase in SSBs was associated with a 17% higher risk of cancer incidence. However, the dose–response association between SSBs and cancer mortality was not studied in that meta-analysis (29). Such a positive association was also reported for cancer mortality in a meta-analysis of 11 observational studies (33). Additionally, a prospective study from Nurses’ Health Study (NHS) reported that women who had SSBs consumption after cancer diagnosis in comparison with women with no consumption had a higher risk of cancer-specific mortality (>1 to 3 serving/week, hazard ratio (HR): 1.31, 95% CI: 1.09–1.58; >3 servings/week, HR: 1.35, 95% CI: 1.12–1.62) (34). Another prospective study from the Iowa Women’s Health Study (IWHS), showed that higher intake of SSBs was positively associated with the risk of type I endometrial cancer (35). In another prospective study among Canadian women, a high intake of sugar-containing beverages was associated with a higher risk of endometrial and ovarian cancers (36). SSBs are considered as a high glycemic (GI) food group. In a meta-analysis of 13 observational studies, after combining data from high-quality studies, we found that adherence to a high-GI diet was positively associated with the risk of endometrial cancer (37). However, in that meta-analysis, SSBs were not assessed.

Despite the mentioned evidence on overall cancer and cancer mortality, data on the link between SSBs and specific cancers are conflicting. A meta-analysis of 27 observational studies reported a significant positive association between consumption of SSBs and risk of breast (RR: 1.14, 95% CI: 1.01–1.30) and prostate cancer (RR: 1.18, 95% CI: 1.10–1.27) (38). However, a meta-analysis and some observational studies reported that intake of SSBs was not significantly associated with pancreatic and colorectal cancers (39–41). This difference might be explained by the influence of IGF-I on the secretion of sex hormones. SSBs have an increasing effect on IGF-I levels. This hormone increases the risk of cancer development through increasing the levels of sex hormones like estrogen and testosterone (42) (Figure 1). The increased levels of the mentioned hormone are the main risk factors for breast and prostate cancers, but not gastrointestinal cancers. Also, differences in adjustments for potential confounders including age, socioeconomic status, smoking, and obesity might be another reason for the observed inconsistency. Among the confounders, obesity has a mediating role because SSBs intake may increase the risk of cancer through increasing energy intake and obesity. Therefore, adjustment for obesity or body mass index (BMI) in some studies may disappear the positive association between SSBs intake and cancer risk.

It should be kept in mind that there is evidence indicating that a higher intake of SSBs among colorectal cancer patients increases the risk of death due to this cancer. For instance, based on a pooled analysis of two cohort studies [NHS and Health Professionals Follow-up Study (HPFS)], each 1 serving/day increase in SSBs was associated with a 59% higher risk of mortality from colorectal cancer (43). In another prospective cohort study from the Cancer Prevention Study-II (CPS-II), higher intake of SSBs was associated with increased risk of colorectal cancer mortality (HR, 1.09; 95% CI, 1.02–1.17; P-trend = 0.011), which remained significant even after controlling for (BMI) (44). Some hypotheses can be proposed for the disparity between colorectal cancer incidence and its mortality in relation to SSBs. Sweetened beverages can increase inflammatory biomarkers through their obesity-induced effects. Also, some studies have shown that the positive association between SSBs and inflammatory biomarkers can occur independently of obesity (45). Given the inflammatory nature of colorectal cancer, SSBs-induced inflammation may adversely affect the prognosis of patients. For other types of cancers such as brain, kidney, and lung cancers, we found no eligible study. Future studies should examine the associations of SSBs with the cancers mentioned in this section.

### Carbonated beverages

Carbonated beverages are common drinks in the world (46). Different types of carbonated beverages have been identified: sugar-sweetened carbonated beverages, not-sweetened carbonated beverages, and those beverages that contain artificial sweeteners. Therefore, some types of these beverages contain a large amount of sugar and therefore have adverse effects on human health (47). Previous studies reported that carbonated beverages consumption was positively associated with dental disease, obesity, and some gastrointestinal diseases such as dyspepsia and gastro-esophageal reflux disease (GERD) (16, 48, 49). Like the SSBs explained in the previous section, those carbonated beverages containing sugar may increase the risk of cancer and its mortality. However, in addition to sugar, other components in the carbonated beverages might be involved (50). One of these probable carcinogen compounds is 4-methylimidazole (4-MI) which is a by-product of the caramel process and coloring agent (51). Experimental studies reported that the high doses of 4-MI were carcinogenic in mice and female rats (52). Therefore, because of this component, not-sweetened carbonated beverages and artificially sweetened beverages without added sugar may be associated with an increased risk of cancer.

The mechanisms mentioned in the previous section are in line with findings obtained from the previous observational studies. The Singapore Chinese Health Study showed that higher consumption of sugar-sweetened carbonated beverages was associated with an 87% higher risk of pancreatic cancer (HR: 1.87, 95% CI: 1.10–3.15) (53). In addition, a pooled analysis of 14 prospective cohort studies showed a modest positive association between sugar-sweetened carbonated soft drinks and the risk of pancreatic cancer (54). A prospective cohort study on middle-aged and older Japanese individuals with stomach cancer indicated that frequent consumption of carbonated drink/juice (RR: 3.9, 95% CI: 1.4–11.1) significantly increased the risk of cancer mortality among women (55). Also, a population-based prospective study in South America showed that a higher intake of sugar-sweetened carbonated beverages was associated with an increased risk of breast cancer-related mortality among cancer patients (56). In contrast, in a case–control study that was conducted in Sweden, a higher intake of carbonated soft drinks (more than six times per week) was not associated with the risk of esophageal adenocarcinoma (57). A prospective study from CPS-II showed no significant association between sugar-sweetened carbonated beverages and the risk of non-Hodgkin lymphoma (NHL) (58). Also, the lack of a significant association between sugar-sweetened carbonated beverages and colon cancer was reported in another study (59). This discrepancy might be explained by the different influences of SSBs on the mentioned tissues. Among the tissues, SSBs have the highest impact on pancreas through increasing insulin production. Also, some studies, that did not show a significant association between SSBs and cancer risk, did not adjust for potential confounding variables including race.

Consumption of sugar-sweetened carbonated beverages is associated with increased blood glucose and hyperinsulinemia. Hyperinsulinemia is associated with an increase in the levels of free IGF-I. Previous evidence has shown that IGF-I increases cell proliferation (60) (Figure 1). In addition, carbonated SSBs contain a large amount of fructose (from the sweetening agent), which can produce advanced glycation end-products. Non-human studies have shown that these products contribute to the development and progression of cancers (61).

Based on our literature search, we found no study investigating not-sweetened carbonated beverages in relation to cancer or its mortality. In terms of artificially sweetened carbonated beverages, a prospective cohort study from CPS-II reported no significant association between daily consumption of artificially sweetened carbonated beverages and the risk of NHL (58). On the other hand, there is evidence of non-carbonated artificially sweetened beverages. In a meta-analysis of 17 prospective studies, Yin et al. concluded that non-carbonated artificially sweetened beverages might be positively associated with the risk of leukemia and negatively associated with the risk of colorectal cancer (62). In another meta-analysis of 38 observational studies, no significant association was reported between artificially sweetened soft drinks and gastrointestinal cancers, particularly colorectal cancer (63). For cancer mortality, no significant association with artificially sweetened soft drinks was reported in a meta-analysis of prospective cohort studies (64). Overall, it seems that findings on the link between artificially sweetened beverages and cancer are conflicting and depend on cancer type. Therefore, since artificially sweetened beverages are consumed in large amounts, future studies should examine the influence of these beverages on all types of cancers.

### Soda

Consumption of soda is increasing in many countries (25). Among the various SSBs choices, sugar-sweetened soda is one of the leading sources of calories and added sugars in Americans’ diets, but it offers nothing else nutritionally (65). Soda might have adverse health effects due to its high sugar content (66). Previous evidence reported that consumption of soda was associated with type 2 diabetes and metabolic syndrome (67).

In terms of cancer, some observational studies have shown that consumption of soda may be associated with an increased risk of cancer. A case–control study that was conducted in South Italy observed that higher consumption of Coca-Cola, as a soda drink, was associated with an increased risk of thyroid cancer (68). Another case–control study in Italy reported a significant positive association between the consumption of cola and the risk of NHL (69). Similarly, a case–control study among the United States (US) population showed a 55% increased risk of pancreatic cancer among patients consuming ≥1 regular cola per day (70). A case–control study from Serbia indicated that the consumption of soda was positively associated with the risk of bladder cancer (71). A pooled analysis of two prospective cohort studies [National Institutes of Health-American Association of Retired Persons (NIH-AARP) Diet and Health Study and the Prostate, Lung, Colorectal and Ovarian Cancer Screening Trial (PLCO)] reported a significant positive association between sugar-sweetened soda consumption and risk of liver cancer (72). Also, a population-based prospective study in South America evaluated the association between sugar-sweetened soda consumption and breast cancer mortality and showed that sugar-sweetened soda drinkers (≥5 times/week) had an 85% increased risk of death due to breast cancer (HR: 1.85, 95% CI: 1.16–2.94) (56). In contrast, in the Multiethnic Cohort Study (MCS), a high consumption of regular soda was not associated with the risk of pancreatic cancer (73). In addition, a retrospective cohort study on the US population, conducted by Davis et al., showed no significant association between cola consumption and pancreatic cancer mortality (70). The controversy observed for pancreatic cancer might be due to different adjustments in the statistical analysis. For instance, the Davis et al. study did not control for energy intake in their analysis. Energy intake is the most important confounders in diet-disease associations (74).

It seems that soda intake is positively associated with both hormonal and non-hormonal cancers. Therefore, other mechanisms, in addition to IGF-I, are involved in the positive associations (Figure 1). Since most sodas are carbonated, these beverages contain high doses of 4-MI which is carcinogenic. In addition, some types of sodas are artificially sweetened. Few studies assessed the association between artificially sweetened sodas and cancer risk. However, findings from these studies are conflicting (72, 75).

### Natural fruit juices

Health promotion and disease prevention guidelines around the world recommend that a variety of fruits and vegetables should be consumed in a day because they contain a wide range of nutrients, particularly fiber and vitamin C (76). Fruit juices are an alternative way to consume sufficient amounts of fruits. Recently, the demand for these types of beverages has increased in many countries (25). Although previous evidence has shown that the consumption of fruits has beneficial effects on health, there is still no agreement on the juices obtained from them (76, 77).

Compared to whole fruit, fruit juices contain a lower amount of fiber and vitamin C. Furthermore, fruit juices are rich in natural sugars and therefore, like SSBs, may have negative effects on human health (78, 79). Hyperglycemia after consumption of juices is associated with increased levels of insulin and IGF-I synthesis, which might enhance tumor development (21). Also, it has been shown that elevated levels of IGF-I have been associated with poor prognosis in cancer patients (21, 22) (Figure 1). Also, fruit juices contain large amounts of fructose, which can produce advanced glycation end-products. These products contribute to the development and progression of cancer tumors (61).

A meta-analysis of 16 prospective cohort studies on 100% fruit juice reported that each 250 mL/day increase in fruit juice intake was associated with a 31% increased risk of overall cancer (RR:1.31, 95% CI: 1.04–1.65), a 22% higher risk of melanoma, a 2% higher risk of squamous cell carcinoma, and a 28% higher risk of thyroid cancer (80). Another meta-analysis of observational studies (11 cohort and 6 case–control studies) indicated that one servings/day increment in consumption of fruit juices was associated with a 14% increased risk of overall cancer (RR: 1.14, 95% CI: 1.06–1.23) and a 32% increased risk of colorectal cancer (33). In a prospective study among Canadian women, a high intake of fruit juice was positively associated with the risk of type I endometrial cancer (36). In addition, a prospective study from the UK Biobank cohort showed a significant positive association between orange juice consumption (>1 serving per day) and the risk of melanoma (81). A positive association between natural fruit juice intake and mortality from breast cancer was reported in a prospective cohort study in the US (82). Similarly, in the European Prospective Investigation into Cancer and Nutrition (EPIC), higher natural juice intake was associated with higher renal cell carcinoma mortality in women (83).

Surprisingly, most studies on natural juices revealed a significant positive association with overall cancer and some specific cancers. However, it should be noted that several cancers such as brain, lung, and renal cancers were not assessed in relation to natural fruit juices. In addition, the types of natural fruit juices were not determined in the previous studies. Natural juices from different fruits may have different effects on cancer risk. There is evidence that some fruit juices such as pomegranate juice have an inverse association with cancer risk (84). Therefore, since the types of antioxidants in fruit juices are different, the influence of these juices on cancer might be different. This should be considered in future studies.

Despite the presence of natural antioxidants in fruit juices, these antioxidants are consumed along with a high amount of natural sugar or fructose. Therefore, it seems that the adverse effects of sugar available in fruit juices cover the beneficial effects of antioxidants.

## Conclusion

In total, we can conclude that most beverages containing natural or added sugar might increase the risk of cancer, particularly sex hormone-related cancers. This positive association might be attributed to IGF-I, which is increased in response to SSBs consumption and induces an increase in sex hormones. In addition to IGF-I, SSBs and natural fruit juices may increase the risk of cancer through their obesity-inducing effects and also their increasing effects on inflammatory biomarkers. Despite the evidence, limited data are available for the link between SSBs/natural fruit juices and some important cancers such as brain, lung, and renal cancers. Therefore, further studies are needed in this regard. In addition, we suggest that a homogenous classification of sweetened beverages should be developed to better understand their roles in the development or maybe prevention of cancer.

## Author contributions

NE: Writing – review & editing, Methodology, Project administration. MZ: Writing – original draft, Methodology. MM: Writing – original draft. ZG: Writing – original draft, Methodology. AA: Writing – original draft. GA: Writing – review & editing, Methodology, Conceptualization. MA: Writing – review & editing, Funding acquisition. AM: Writing – review & editing, Conceptualization. OS: Writing – review & editing, Supervision.
